# Immobilization protects enzymes from plasma-mediated inactivation

**DOI:** 10.1098/rsif.2023.0299

**Published:** 2023-10-25

**Authors:** Tim Dirks, Abdulkadir Yayci, Sabrina Klopsch, Marco Krewing, Wuyuan Zhang, Frank Hollmann, Julia E. Bandow

**Affiliations:** ^1^ Applied Microbiology, Faculty of Biology and Biotechnology, Ruhr University Bochum, Bochum, Germany; ^2^ National Innovation Center for Synthetic Biotechnology, Tianjin Institute of Industrial Biotechnology, Chinese Academy of Sciences, 32 West 7th Avenue, Tianjin Airport Economic Area, Tianjin 300308, People's Republic of China; ^3^ Department of Biotechnology, Delft University of Technology, Delft, The Netherlands

**Keywords:** atmospheric plasma, non-thermal plasma, immobilization, enzyme protection

## Abstract

Non-thermal plasmas are used in various applications to inactivate biological agents or biomolecules. A complex cocktail of reactive species, (vacuum) UV radiation and in some cases exposure to an electric field together cause the detrimental effects. In contrast to this disruptive property of technical plasmas, we have shown previously that it is possible to use non-thermal plasma-generated species such as H_2_O_2_ as cosubstrates in biocatalytic reactions. One of the main limitations in plasma-driven biocatalysis is the relatively short enzyme lifetime under plasma-operating conditions. This challenge could be overcome by immobilizing the enzymes on inert carrier materials. Here, we tested whether immobilization is suited to protect proteins from inactivation by plasma. To this end, using a dielectric barrier discharge device (PlasmaDerm), plasma stability was tested for five enzymes immobilized on ten different carrier materials. A comparative analysis of the treatment times needed to reduce enzyme activity of immobilized and free enzyme by 30% showed a maximum increase by a factor of 44. Covalent immobilization on a partly hydrophobic carrier surface proved most effective. We conclude from the study, that immobilization universally protects enzymes under plasma-operating conditions, paving the way for new emerging applications.

## 1. Introduction

Non-thermal plasmas are gaining popularity in the biological sciences, with applications in surface sterilization [1], wound disinfection [2], cancer treatment [3,4], agriculture [5,6], and white biotechnology [7]. While the field of application is diverse, the underlying principle typically is similar: it is mainly the reactive oxygen and nitrogen species (RONS) generated in the plasma that interact with biological matter causing desired reactions [8]. Biological samples generally have a high water content and in some applications the samples are fully submersed in aqueous liquids. Plasma exposure thus not only entails plasma species but also reaction products generated at plasma–liquid interfaces and downstream in the bulk liquid [9]. On a molecular level, these reactions have been shown to inactivate a variety of biomolecules, including DNA [10,11], lipids, cell envelope components [12–14], and, most prominently, proteins [15–17].

Protein inactivation by plasmas is well documented and has been attributed mainly to the reaction of plasma-generated RONS with proteins. The inactivation mechanisms are manifold but two inherently different plasma-protein interactions can be differentiated. Chemical modifications of amino acid side chains within a protein can result from interactions with short-living or long-living RONS. Depending on the function of the modified amino acid, this can lead to inactivation that is due to protein unfolding [18–20] or to the modification of catalytically active amino acids [15]. Besides side chain modifications, the cleavage of the protein backbone has been observed. In this case, the peptide bonds are cleaved resulting in an increase in terminal amino functions. This inactivation mechanism is mainly attributed to rather short-living plasma species such as hydroxyl radicals [21,22]. Furthermore, RONS can also damage cofactors or prosthetic groups, irreversibly inactivating proteins that are cofactor-dependent and require these for protein activity [23–25].

For certain applications, such as surface sterilization, inactivation of proteins is a desired mechanism and thus promoted. However, for other applications, such as plasma-driven biocatalysis, it is crucial that proteins are protected from inactivation. In plasma-coupled biocatalysis, plasma-generated hydrogen peroxide (H_2_O_2_) is used as cosubstrate to convert organic substrates into more valuable products [7]. Although H_2_O_2_-dependent enzymes require a certain H_2_O_2_ level to operate, they are inactivated by high concentrations of H_2_O_2_ as well as by other RONS. Since enzymes significantly contribute to biocatalysis cost, success depends on a long enzyme lifetime that is achieved by an effective control of the levels of reactive species that come in contact with the enzymes [7]. In previous works, we identified protein immobilization as a highly effective strategy to protect enzymes from plasma-mediated inactivation [26]. These studies exclusively focused on unspecific peroxygenase from *Agrocybe aegerita*, an enzyme with biotechnological potential that was selected to prove that plasma-driven biocatalysis is possible [27,28].

**Figure 1.**
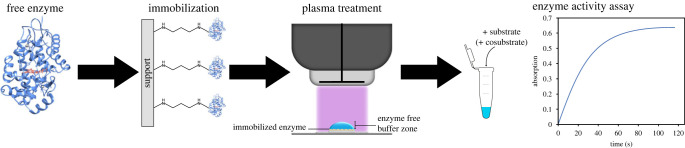
Schematic workflow of the study. Free enzyme was immobilized on different carrier materials (Lifetech ECR resins or EziG beads) via different functional groups. After immobilization, the enzyme-containing liquid was treated with a DBD device. Afterwards, enzyme activity was determined in a decoupled assay. The HRP model is based on the PDB 1HCH entry.

The aim of the present study was to test whether immobilization is a more broadly applicable strategy to protect enzymes from plasma-mediated inactivation, independent of plasma-driven biocatalysis or the requirement of H_2_O_2_ as cosubstrate. Five enzymes were selected, two of which, like the enzymes used in plasma-driven biocatalysis, are H_2_O_2_-dependent, namely horseradish peroxidase (HRP) and vanadium chloroperoxidase (VCPO), while the other three enzymes (lactate dehydrogenase (LdhA), beta-galactosidase (LacZ) and glyceraldehyde 3-phosphate dehydrogenase (GapA)) do not require H_2_O_2_ or cofactors. In previous studies, HRP and GapA enzymes have been shown to be rapidly inactivated by plasma treatment [7,10]. Therefore, they lent themselves to test for plasma-protective effects of immobilization. To investigate a potential benefit of immobilization on enzyme stability under plasma-operating conditions, we compared inactivation rates of free and immobilized enzyme in an aqueous solution. To this end we treated the samples with a dielectric barrier discharge (DBD) device (PlasmaDerm from Cinogy) and, after the plasma treatment, evaluated the residual enzyme activity in decoupled, enzyme-specific activity assays (figure 1). Several immobilization strategies were compared, and, in all cases, immobilization significantly increased enzyme lifetime under plasma treatment conditions.

## 2. Material and methods

### 2.1. Enzymes

HRP was purchased from Sigma-Aldrich (P8375, *RZ* > 2.5) and stored in 100 mM potassium phosphate buffer (KPi), pH 7. The enzymes LdhA, LacZ and GapA were purified as N-terminal His_6_-tag fusion proteins from *E. coli* BL21 strains harbouring the plasmids pCA24N::*ldhA*, pCA24N::*lacZ*, and pCA24N::*gapA*, respectively [29]. Cells were grown in LB medium containing 50 µg ml^−1^ chloramphenicol [29]. For overexpression, overnight cultures were diluted to an OD_600_ of 0.05 and incubated at 37°C. At OD_600_ of 0.5, overexpression was induced with 100 µM isopropyl β-D-thiogalactoside (IPTG). Cultures were shifted to 30°C and harvested after 4 h of growth following the shift. Cell pellets were resuspended in lysis buffer (20 mM sodium phosphate, 500 mM NaCl, 0.2 mg ml^−1^ DNase, 0.2 mg ml^−1^ RNase, 0.35 mg ml^−1^ lysozyme, cOmplete protease inhibitor (Roche); all other components acquired from Sigma-Aldrich). After sonication and centrifugation for 30 min (21 000*g*), the supernatant was loaded onto a HisTrap FF crude 5 ml column (GE Healthcare). Purification of the His_6_-tagged proteins was carried out with an ÄKTA pure25 system (GE Healthcare). Proteins were eluted with a linear gradient from 20 to 500 mM imidazole in 20 mM sodium phosphate buffer and 500 mM NaCl. Elution fractions of interest were pooled, concentrated using centrifugal filter units (cut-off 10 kDa), aliquoted, and stored in potassium phosphate buffer (100 mM, pH 7) at −80°C.

The expression plasmid for His_6_-VCPO was constructed by amplifying the native *Curvularia inaequalis* VCPO gene from pBAD::*vcpo* by PCR [30], using the following primers: forward, 5-AAAAGCTAGCATGGGGTCCGTTACACCCATCC-3; reverse, 5-AAAAGGATCCCTACGGCGCCTCCTTGACTACC-3. The PCR product was ligated with pET28b using the *Nhe*I and *Bam*HI sites. The finalized construct was transformed into *E. coli* BL21 and used for overexpression as described above with minor changes: cells were grown in LB medium containing 100 µg ml^−1^ kanamycin, and Tris sulfate buffer (50 mM, pH 8) was used for purification and storage instead of phosphate buffer. Prior to plasma treatment, His_6_-VCPO was subjected to buffer exchange to potassium phosphate buffer (pH 7).

### 2.2. Plasma treatment

Droplets of protein samples (containing either 100 µl of 1 mg ml^−1^ free enzyme or 100 µl with 10 mg protein-loaded ECR carrier or 0.5 mg protein-loaded EziG carrier) were applied onto a glass slide partially coated with polytetrafluoroethylene (PTFE; supplier: VWR), such that the droplets sat on glass and were confined by the PTFE coating. The glass slide was placed onto a grounded metal plate. Plasma treatment of the samples was carried out using the PlasmaDerm dielectric barrier discharge (DBD) (Cinogy) with an electrode diameter of 20 mm, *V*_RMS_ = 13.5 kV, and a trigger frequency of 300 Hz. The distance of the sample to the driven electrode was approx. 1 mm (± 1 mm). For experiments using Ni-NTA agarose as immobilization carrier, plasma treatment was carried out with in-house built metal plates (electronic supplementary material, figure S1), which are formed in such a way that they can be placed on reaction tubes in place of the lid. This allowed for complete recovery of the samples by centrifugation.

### 2.3. Immobilization

For immobilization, two different types of carriers were used, namely non-directional Lifetech ECR resins (Purolite ECR1 kit) and directional EnginZyme (EziG) supports. For all Lifetech resins, 200 mg of resin were weighed into a suitable vessel and washed thrice with potassium phosphate buffer (KPi) (100 mM, pH 7). Additionally, the amino-functionalized resin was incubated with 0.4% glutaraldehyde in KPi for 1 h and washed thrice in KPi. Afterwards, enzymes (2 nmol) were added to the carriers and allowed to bind during an incubation for 16 h at 8°C with overhead shaking. The supernatant was removed and the binding efficiency determined. To this end, the activity of the supernatant was subtracted from the activity of the enzyme solution initially used for immobilization to yield the binding efficiency in %. All enzyme activities were determined based on the initial slope of the reaction and the activity of free enzyme is assumed to be directly proportional to the amount of enzyme (electronic supplementary material, figure S2, tables S1–S5). Before further analysis, the beads were washed thrice again using KPi.

For EziG carriers, 10 mg of Opal (EziG1), Amber (EziG2), or Coral (EziG3) were washed thrice using KPi. Afterwards, 2 mg of His_6_-fusion enzymes were added in a total volume of 2 ml and incubated for 16 h at 8°C with overhead shaking. The supernatant was removed, and the beads were washed thrice with KPi. Binding efficiency was calculated as described for the non-directional resins (electronic supplementary material, figure S2, tables S6–S9).

### 2.4. Activity assays

Enzyme activities were determined in a total volume of 200 µl using equal volumes of substrate-containing buffer and reaction starting agent (substrate and if applicable cosubstrate). Enzyme kinetics were monitored using a microplate reader (Biotek Epoch). Activity was calculated based on the linear slope of the reaction.

Final concentrations and the respective wavelengths at which reactions were monitored are as follows. For HRP, 0.1 U ml^−1^ protein was used with 2.5 mM 2,2′-azino-bis(3-ethylbenzothiazoline-6-sulfonic acid) (ABTS) (*λ* = 405 nm), 50 mM citrate buffer (pH 5), and 1 mM H_2_O_2_. For VCPO, 375 nM protein, 0.2 mM phenol red (*λ* = 582 nm), 0.05 mM sodium orthovanadate, 5 mM KBr, 50 mM tris sulfate buffer (pH 8), and 5 mM H_2_O_2_ were used. The LdhA reaction was measured with 685 nM protein, 20 mM sodium pyruvate, 100 mM KPi (pH 7), and 0.25 mM β-nicotine amide adenine dinucleotide disodium salt (NADH) (*λ* = 340 nm), LacZ activity with 10 nM protein, 10 mM sodium chloride, 1 mM MgSO_4_, 100 mM KPi (pH 7), and 2 mM 2-nitrophenyl-β-D-galactopyranosid (ONPG) (*λ* = 420 nm). GapA activity was determined with 700 nM protein, 0.625 mM glyceraldehyde 3-phosphate (GAP), 100 mM KPi (pH 7), and 0.625 mM β-nicotine amide adenine dinucleotide (NAD^+^) (*λ* = 340 nm).

Activities of immobilized enzymes were determined as described for the free enzymes, but in a total volume of 1 ml containing 10 mg of protein-loaded ECR carriers or 0.5 mg of protein-loaded EziG carriers. Protein concentrations for each respective enzyme and immobilization carrier are given in electronic supplementary material, tables S1–S9. Samples were shaken during turnover to ensure sufficient substrate supply. Over the course of a 10 min reaction time, every two minutes a 100 µl aliquot was taken from the reaction solution to immediately measure the respective wavelengths using a microplate reader (Biotek Epoch). Withdrawal of aliquots does not affect the enzyme reaction in the remaining volume. Enzyme activity was calculated based on the linear slope of the kinetics. For each enzyme–carrier pair, raw activities determined in the activity assays and specific activities (activities normalized to protein concentration) are given in electronic supplementary material, figure S3. Relative specific enzyme activities are reported in relation to untreated protein on the same carrier.

### 2.5. Reversible immobilization

For reversible immobilization, 10 µl Ni-NTA agarose (Qiagen) were washed three times with KPi (100 mM, pH 7) and incubated with 40 µl of 1 mg ml^−1^ enzyme for 16 h at 8°C. Samples were briefly centrifuged at 15 500*g* and the supernatant was removed. The concentration of unbound protein in the supernatant was determined using the Bradford method. The Ni-NTA agarose was washed three times with KPi and dissolved in 40 µl buffer. Before plasma treatment, samples were incubated for 5 min on the metal plates used for plasma treatment to allow the agarose resin to settle. To investigate potential enzyme detachment from the agarose upon plasma treatment, the protein concentration in the supernatant was measured again after plasma exposure. Subsequently, proteins were eluted from the agarose with 20 mM sodium phosphate buffer containing 500 mM NaCl and 500 mM imidazole. Eluted protein was then washed with KPi and concentrated using centrifugal filter units (cut-off 10 kDa). Finally, the concentration of the eluted protein was measured, and enzyme activity was determined as described above. Enzyme activities are given as specific activities (raw activity per milligram of protein). Specific activities of the untreated enzymes were set to 100%.

### 2.6. Quantitation of amino groups

Quantitation of terminal amino groups provides a readout for protein degradation and was carried out using ninhydrin as described elsewhere [22]. Free enzyme or reversibly immobilized enzyme was treated with plasma for different amounts of time, and immobilized enzyme was then eluted before determining the concentration of N-termini.

## 3. Results and discussion

**Figure 2.**
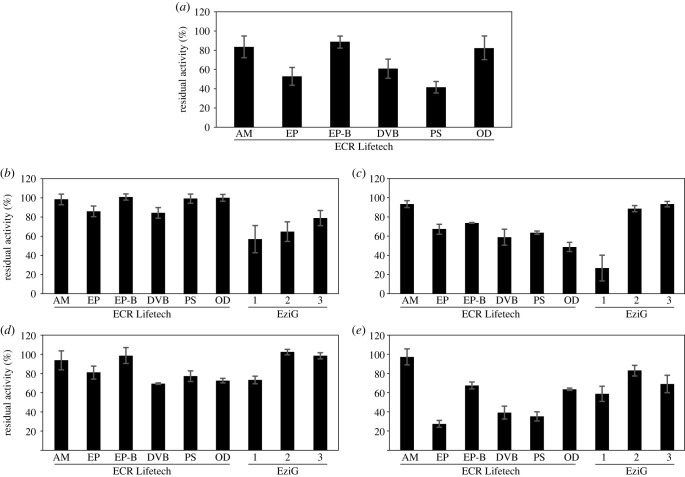
Residual activity of immobilized HRP (*a*), VCPO (*b*), LdhA (*c*), LacZ (*d*), and GapA (*e*) after 900 s of plasma treatment (180 s for GapA). Immobilization was carried out using Lifetech ECR (Purolite) and EziG (EnginZyme) carriers according to the manufacturers' instructions. For HRP, only non-directional resins were used for immobilization, because the enzyme did not have a His-tag. The specific activity of the respective untreated immobilized enzyme was set to 100%. Means and standard deviations of three independent biological replicates are shown. Bead types are abbreviated as follows: AM: amino, EP: epoxy, EP-B: epoxy-butyl, DVB: divinylbenzene, PS: polystyrene, OD: octadecyl.

In previous works, we have demonstrated that immobilization protects H_2_O_2_-dependent unspecific peroxygenase from plasma-mediated inactivation. While those works were limited to that enzyme alone, here we set out to evaluate the broader scope of protection effect using a panel of enzymes from different classes that were each immobilized using various strategies. Besides the two hydrogen peroxide-dependent enzymes HRP and VCPO, the hydrogen peroxide-independent enzymes LdhA, LacZ and GapA were tested. For the commercial HRP, only non-directional immobilization was used due to the missing His_6_-tag. Aliquots of immobilized enzyme were treated with a DBD plasma in ambient air. The activity of untreated immobilized enzyme was set to 100%. Based on previous studies investigating the inactivation rates of free enzymes under plasma-operating conditions using the DBD device, residual enzyme activities were determined after 900 s of plasma treatment [7,10,26] (figure 2). Plasma treatment times for GapA were reduced to 180 s, since this enzyme proved to be comparably sensitive to inactivation.

In general, amino- and epoxy-butyl-functionalized immobilization provided the highest level of plasma protection for all enzymes. Moreover, the covalent immobilization methods (amino, epoxy and epoxy-butyl) of the non-directional resins offered greater protection against plasma-mediated enzyme inactivation than the non-covalent methods (DVB, polystyrene and octadecyl). This might be explained by the greater rigidity in the enzyme structure induced by covalent immobilization [31,32]. The epoxy-butyl-functionalized immobilization method showed higher residual activities for all enzymes in comparison to the epoxy-based method, indicating that the more hydrophobic surface provides a higher level of protection against plasma. This effect was also observed in previous works [26] and is in line with the results of the directional immobilization supports (EziG1–3). Overall, enzyme lifetimes were higher when proteins were immobilized on the more hydrophobic surfaces of EziG2 or EziG3 compared to EziG1. As postulated previously [26], a more hydrophobic surface of the carrier materials may help to repel hydrophilic ROS and RNS from interaction with the enzyme. However, this beneficial effect only seems to arise in combination with covalent immobilization, since the hydrophobic, non-covalent resins (DVB, polystyrene and octadecyl) presented lower levels of plasma protection.

**Figure 3.**
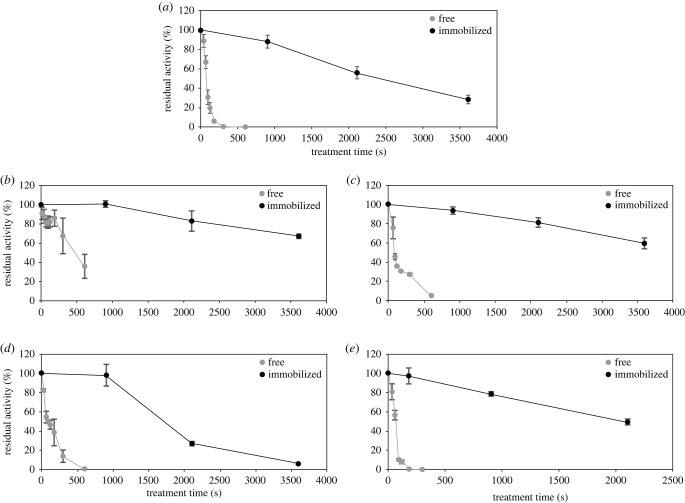
Residual activities of free enzyme and enzyme immobilized on the most efficiently protecting carrier material (either amino or epoxy-butyl based Lifetech ERC resins (Purolite)) after different plasma treatment times. HRP immobilized on epoxy-butyl (*a*), VCPO on epoxy-butyl (*b*), LdhA on amino (*c*), LacZ on epoxy-butyl (*d*), and GapA on amino (*e*) based ERC resin. Plasma treatment times of up to 3600 s were used (note different x-axes ranges). The specific activity of untreated enzyme was set to 100% activity. Data of free HRP are taken from Yayci *et al.* [7]. Means and standard deviations of three independent biological replicates are shown.

The protective effect of immobilization against plasma-mediated enzyme inactivation was originally thought to be because of a buffer zone between the enzymes and the liquid surface presenting the entry point of plasma-produced species. The aqueous buffer zone allows for short-living plasma species, which are thought to be most harmful to proteins [22], to recombine before reaching the immobilized enzyme. Since the different types of carriers differ in the extent to which they protect the five enzymes from activity loss, additional effects must be at play. The carrier material could act as scavenger through interactions with plasma-derived species, e.g. through free functional groups, preventing the interaction of plasma species with the enzymes. Here, different characteristics of the carrier materials and different functional groups could explain different levels of plasma protection of the enzymes. The results for GapA, for instance, highlight the influence of the carrier. Immobilization of GapA on amino-functionalized material showed 50% residual activity after 2100 s of plasma treatment (figure 3), while for any other carrier almost complete inactivation was reached after 900 s of plasma treatment (electronic supplementary material, figure S4).

To quantify the level of protection provided to the enzymes by immobilization, residual activity after plasma treatment was compared for free and immobilized enzymes (figure 3). For each enzyme, data for the carrier material with the highest level of plasma protection are shown (figure 2; electronic supplementary material, table S10).

**Figure 4.**
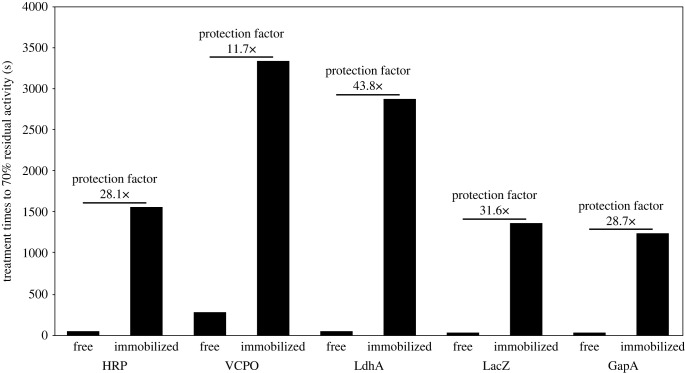
Treatment times at which residual enzyme activity of free and immobilized HRP, VCPO, LdhA, LacZ and GapA reached 70%. HRP, VCPO, and LacZ were immobilized on epoxy-butyl-functionalized resin, LdhA and GapA on amino-functionalized resin following manufacturers' instructions. The treatment time at which residual activity reached 70% was calculated based on the respective inactivation curve regression.

For all free enzymes except VCPO, complete inactivation was detected after 600 s of plasma treatment. GapA proved particularly plasma sensitive and was inactivated completely after 300 s treatment. This was to be expected based on previous reports on GapDH, the homologous protein from rabbit muscle, which was quickly inactivated by plasma, likely due to oxidation of the catalytic cysteine residue in the active site of the protein [10]. Even for the immobilized GapA, treatment times of only up to 2100 s were tested due to the more rapid inactivation, while all other immobilized enzymes were treated for up to 3600 s. For all five proteins, inactivation was markedly slower for the immobilized compared to the free enzyme. All immobilized proteins except LacZ still showed considerable activity after the longest treatment time. Activities of VCPO and LdhA did not drop below 50% within 3600 s. Nonetheless, immobilized enzymes also suffered inactivation after prolonged exposure. Under these conditions, inactivation may be attributed at least partially to long-lasting RONS like H_2_O_2_, ONOO^−^, or acidification (H^+^) which are known to cause enzyme inactivation over time, but at a significantly lower rate than short-living species (which likely react to less reactive species in the buffer zone). To compare protective features across resins and proteins, we determined protection factors. To this end for free and immobilized enzyme the treatment times leading to a 70% residual enzyme activity were calculated based on the respective inactivation curve regression (figure 4). 70% residual activity was chosen for comparing the effects on different enzymes for practical reasons: (1) we were limited by plasma operation time to 1 hour and (2) for the most stable enzyme under plasma-operating conditions (VCPO), the lowest residual enzyme activity detected after 1 h was approx. 65%.

For HRP, LacZ, and GapA, immobilization resulted in an approximately 30-fold increase in treatment times at which protein activity was reduced to 70%, while for LdhA the increase was approximately 44-fold. For VCPO the treatment time required was the longest; however, due to the comparably high plasma stability of the free enzyme, the protection factor was only 12. For all five enzymes, a great increase of enzyme lifetime was observed, so that we can conclude that immobilization is a universal strategy to protect enzymes from detrimental effects of plasma treatment.

Significant differences in residual enzyme activity were observed for enzymes immobilized on different supports that were treated with plasma (figure 2). We were interested in how far the functional groups on the carrier material might influence the supply with different substrates. The enzymes investigated in the present study all convert rather hydrophilic organic substrates: ABTS (HRP), Cl^−^, the reaction product of which, OCl^−^, then spontaneously reacts with phenol red (VCPO), sodium pyruvate (LdhA), ONPG (LacZ), and GAP (GapA). To investigate whether—independent from plasma treatment—the hydrophobicity of the substrates and the carrier material influence enzyme activity measurements, skewing the determination of residual activities of different enzyme–carrier pairs under plasma treatment conditions, we investigated an enzyme that can convert both hydrophilic and hydrophobic substrates. We determined the residual activity for the unspecific peroxygenase r*Aae*UPO as described previously, using either the hydrophilic substrate ABTS [26] or the hydrophobic substrate ethylbenzene (ETBE). Specific activity after plasma treatment was the same for both substrates (electronic supplementary material, figure S5), indicating that at least for that model protein, the substrate does not influence the determination of the residual activity after plasma treatment.

**Figure 5.**
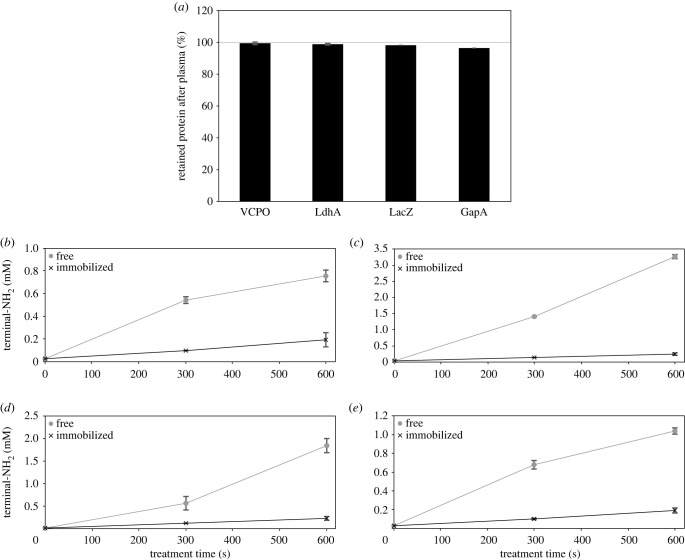
Plasma-mediated protein detachment from the carrier material and protein degradation under plasma treatment conditions were investigated using Ni-NTA agarose, to which enzyme is bound non-covalently. Proteins VCPO, LdhA, LacZ, or GapA were immobilized on Ni-NTA agarose and treated with plasma or left untreated. (*a*) After 300 s of plasma treatment, the amount of protein in the supernatant was determined using a Bradford assay and compared to protein concentrations in the untreated control. (*b–e*) Using ninhydrin, after plasma treatment times of up to 600 s, the concentration of terminal amino groups as an indicator of protein degradation was determined for free enzyme and enzyme immobilized on Ni-NTA agarose: VCPO (*b*), LdhA (*c*), LacZ (*d*), and GapA (*e*). Means and standard deviations of three independent biological replicates are shown.

We were further interested to determine the utility of the non-covalent carrier Ni-NTA by addressing the potential release of protein from the carrier, protein protection from degradation and from inactivation. His_6_-tagged enzymes were reversibly immobilized on Ni-NTA agarose prior to treatment with plasma, and then protein concentrations and concentrations of N-termini were determined in the supernatant, and protein activity measured for enzyme recovered from the carrier. The increase in N-termini was used to estimate protein degradation attributable to backbone breakage. Further plasma-mediated oxidation of the resulting amino groups may not allow a precise detection of all cleavage events. However, by comparing plasma-mediated cleavage products of different proteins to products of protein degradation by acid hydrolysis, Krewing *et al.* demonstrated that after plasma treatment more than 70% of the maximally possible amino groups can be detected in the ninhydrin assay [22]. Comparing protein amounts in supernatants of treated and untreated Ni-NTA-bound protein, no significant differences were detected (figure 5*a*). This showed that proteins are not detached from the carrier by the plasma treatment. The immobilization on Ni-NTA provided protection from plasma-mediated degradation to all enzymes (figure 5*b–e*), albeit to different extent. After 300 s of plasma treatment, the increase in the number of N-termini was comparably high for VCPO (30% compared to free enzyme control), which had a comparably low degradation rate even for the free enzyme. LacZ, which as free enzyme was readily degraded upon plasma treatment, was efficiently protected by immobilization—the concentration of N-termini increased to only 12% of the free enzyme control. The results confirm that enzyme immobilization more broadly protects from protein degradation by plasma, which was previously shown for r*Aae*UPO [26]. It is mainly the short-living species that play a key role in plasma-induced protein degradation. Short-living plasma species are thought to recombine to form long-living species as shown in simulations and by spectroscopic measurements for instance for atomic oxygen (short-living species) which recombines in the effluent of a cold atmospheric pressure plasma jet (COST jet) to form ozone (long-living species) [33,34]. By analogy with the observed and simulated reactions of the effluent of plasma jets, simulations of the reactions occurring in a DBD plasma suggest similar effects [35]. In addition, several simulations suggest that hydroxyl radicals, a species considered to be the key species in protein degradation, may recombine to form the long-lived species hydrogen peroxide [36–39]. Creating an enzyme-free buffer zone allows the highly reactive short-living species to recombine before they reach the enzyme, protecting enzymes from plasma-induced degradation.

**Figure 6.**
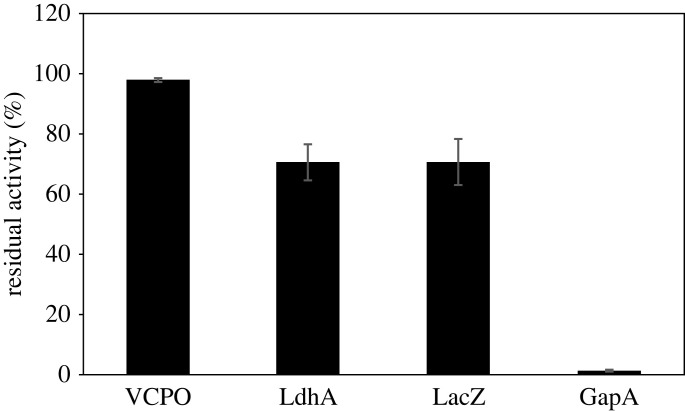
Residual activity of VCPO, LdhA, LacZ, or GapA reversibly immobilized on Ni-NTA agarose after 300 s of plasma treatment. After plasma treatment, enzyme was eluted from the carrier with imidazole and washed with buffer. Protein concentration was determined using Bradford assay. Enzyme activity was measured using 5 µl of the eluted enzyme in its respective activity assay. Activities were normalized to the respective protein concentrations and activity of the untreated enzyme was set to 100%. Means and standard deviations represent three independent biological replicates.

After plasma treatment, proteins were eluted from the Ni-NTA agarose, and protein concentration and enzyme activity determined. The activity of the eluted, but untreated enzyme was set to 100% (figure 6), and all activities were normalized to the respective protein amount. As previously observed for the non-reversible carriers, the immobilized enzymes showed higher activity after 300 s of plasma treatment than the respective free enzymes. For VCPO, essentially no inactivation was detected (97% residual activity), while LdhA and LacZ residual activities were approximately 70%. GapA was almost completely inactivated, with 2% residual activity. These results confirm a high variation in plasma stability of different enzymes. It also highlights the great differences observed for different enzyme–carrier pairs: GapA activity was protected much more efficiently when immobilized covalently on a butyl-functionalized ERC resin (figure 3*e*) than non-covalently on Ni-NTA agarose (figure 6).

We conclude that reversible immobilization provided good protection from plasma-mediated degradation, but different levels of protection from enzyme inactivation, likely depending on the inactivation mechanism. Proteins such as GapA, that are inactivated by long-living species (H_2_O_2_) [40,41], suffer inactivation even when immobilized. The experiment with r*Aae*UPO indicated that the hydrophobicity of the substrate did not affect the determination of enzyme residual activity (electronic supplementary material, figure S5). The mechanisms underlying enzyme protection by immobilization remain to be elucidated, and they might differ between different enzyme–carrier pairs.

In summary, this work evaluated the protective effects of enzyme immobilization under plasma-operating conditions using different enzymes and immobilization methods. The covalent immobilization on a hydrophobic carrier surface provided the highest level of protection. All tested enzymes showed highest plasma stability on the amino- or epoxy-butyl-functionalized carriers, making them the most promising carriers for plasma-driven biocatalysis. While immobilization effectively protects all enzymes from short-living species, some proteins are still inactivated by long-living RONS. Therefore, future protection strategies based on immobilization will further need to encompass enzyme-specific protection from long-living RONS.

## Data Availability

The data are provided in electronic supplementary material [42].
